# Cardiovascular Disease and Diabetes: A New Challenge in the Treatment and Management

**DOI:** 10.3390/ijms27010354

**Published:** 2025-12-29

**Authors:** Graziano Riccioni, Chiara Notarangelo, Mario Riccioni, Nicolantonio D’Orazio

**Affiliations:** 1 Cardiology Care Unit, “San Camillo de Lellis” Hospital, 71043 Foggia, Italy; 2 Department of Cardiovascular Disease, University of Firenze, 50134 Florence, Italy; notarangelochiara4@gmail.com; 3 Faculty of Medicine, University of Torino, 10126 Turin, Italy; mmrr1640@gmail.com; 4 Department of Biomedical Sciences, University of Pescara-Chieti, 66100 Chieti, Italy; ndorazio@unich.it

**Keywords:** cardiovascular disease, diabetes, metformin, SGLT2 inhibitors, GLP-1 receptor antagonists, DDP-4 inhibitors, prevention

## Abstract

Cardiovascular diseases (CVDs) represent one of the leading causes of morbidity and mortality in patients with diabetes. However, a correct and effective glycaemic control obtained by pharmacologic interventions, such as the use the novel glucose-lowering agents, demonstrated efficacy in reducing the risk of both cardiovascular events and mortality. The latest classes of glucose-lowering drugs introduced in the clinical practice are DPP4 inhibitors (sitagliptin, saxagliptin, vildagliptin, linagliptin, and alogliptin), GLP-1 receptor agonists (semaglutide, liraglutide, albiglutide, dulaglutide, exenatide, and lixenatide), and SGLT-2 inhibitors (empaglifozin, canaglifozin, and dapaglifozin). Multiple lines of evidence show that all these new drugs associated with the treatment of diabetic disease have the same effectiveness as the traditional antidiabetic drugs, and excellent cardiovascular safety, highlighting their potential in significantly reducing major cardiovascular events and mortality. The aim of our review is to summarise the clinical efficacy of these recently introduced drugs to optimise treatment strategies, especially in the early phase of diabetic disease.

## 1. Introduction

T2D (type 2 diabetes mellitus) is one of the main risk factors for developing cardiovascular disease (CVD), with manifestations including coronary artery disease (CAD), heart failure (HF), stroke, aortic and peripheral artery diseases (PAD), and chronic kidney disease (CKD) [1].

The combination of T2D with these cardio-renal comorbidities enhances the risk not only of cardiovascular (CV) events but also of CV and all-cause mortality [2].

Framingham observations, for the first time, revealed that T2D could be linked to the development of CVD, because patients with T2D had a three-fold increased risk, and these observations were confirmed by numerous later studies [3]. In addition, patients with T2D and HF, whether with reduced ejection fraction (HFrEF), or mid-range (HFmrEF) or preserved ejection fraction (HFpEF), present a significant increase in morbidity and mortality [4]. Overall, the prognosis of CVD in patients with T2D seems to be worse when compared to that of the general population.

Important issues regarding the management of patients with T2D and CVD are as follows:

1. An interdisciplinary healthcare clinician approach from different disciplines and expertise in several medical areas;
2. A personalised treatment strategy to reduce each patient’s disease burden, resulting from the presence of cardio-nephro-metabolic complications which modify therapeutic indications;
3. Common goals in managing CVD in patients with T2D to improve patients’ prognosis and health-related quality of life.

The American and European Guidelines emphasise the key role of diet and lifestyle changes in preventing T2D and its CV complications [2,5]. Weight loss in obese patients with T2D could be needed to reduce the risk of CVD and improve insulin sensitivity [6].

The Look AHEAD Research Group examined the effect of intensive lifestyle intervention for weight loss, finding decreased CV morbidity and mortality in patients with T2D; even if there were no differences in terms of mortality and morbidity between the intervention and the control group, the first one registered a greater reduction in glycated haemoglobin (HbA1c) and a greater improvement in all CV factors [6]. 

Other important health outcomes such as nephropathy were analysed: data from this trial showed that the incidence rate of very-high-risk chronic kidney disease (CKD) was 31% lower in the intervention group compared to the control one and this effect was attributable in part to reductions in weight, HbA1c, and blood pressure [7]. Moreover, the Mediterranean diet, which includes vegetables, fruits, legumes, and polyunsaturated and monounsaturated fats, should be preferred because it reduces the incidence of major CV events, as shown in the Prevention con Dieta Mediterranea (PREDIMED) study, which involve people at high CV risk (49% in patients with T2D) [8].

Physical activity should be implemented because of its crucial impact on glycaemic control and cardiovascular well-being [9]. However, changing lifestyle is not always enough and therefore, pharmacological agents can be helpful to optimise treatment strategies and to gain an adequate glycaemic control.

Several clinical trials revealed that controlling blood glycaemic levels drives a significant decrease in CV events, especially in patients affected by HF. A meta-analysis of three major studies, ACCORD, ADVANCE, and VADT, showed that a reduction in HbA1c of ≥1% is associated with a 15% relative risk reduction in non-fatal MI. Patients who benefit most from intensive glucose control are those with a short duration of T2D, lower HbA1c, and no CVD. Therefore, glucose control in younger individuals or in patients with a recent diagnosis of DM should be commenced as soon as possible to prevent cardiovascular events, while in elderly patients it is possible to establish less-rigorous targets [2].

Recently, the results of various large CV outcome trials (CVOTs) [10] in patients with T2D at high CV risk treated with novel glucose lowering agents, such as Dipeptidyl peptidase 4 inhibitors (DPP4-I), sodium–glucose co-transporter-2 (SGLT-2) inhibitors and glucagon-like peptide-1 (GLP-1) receptor agonists (RAs), substantially expanded available therapeutic options, leading to numerous evidence-based recommendations for the management of this patient population [2,10]. It is important to establish an optimal treatment, considering both the most glycaemic target and the most favourable benefit/safety ratio. Consequently, since 2008, several CV trials were conducted to study the CV safety of all new antidiabetic drugs. The efficacy and tolerability profiles of these therapeutic agents, evaluated in different trials, both retrospective and prospective, will be described below.

## 2. Efficacy—Estabilished Oral Glucose-Lowering Drugs

### 2.1. Metformin

Metformin is among the drugs used as a first line of treatment in diabetic diseases. Controlling blood sugar is achieved by reducing glucose production in the liver and activating AMP-activated protein kinase (AMPK), which has multiple functions in addition to regulating the energy homeostasis of cells [11]. In fact, it also regulates the production of endothelial nitric oxide synthase (eNOS), which has protective effects [12,13]. Additionally, metformin reduces oxidative stress, inflammatory response, and platelet aggregation [14,15]. Thus, by acting on multiple targets besides glycaemic ones, it results in several positive effects on the CV system that are the subject of different prospective studies [16].

### 2.2. Thiazolinedion

Thiazolidinediones are effective drugs that reduce insulin resistance by activating the peroxisome gamma receptor (PPAR-γ), which controls the expression of key genes for cell energy homeostasis and is located within adipose tissue, muscle, and liver [17]. This class of drugs is not only able to increase insulin sensitivity, achieving a better glycaemic control, but is also able to reduce the level of circulating triglycerides and cholesterol low density lipoprotein (C-LDL) and oxidative stress [18]. Therefore, all of these factors seem to be effective in reducing CV risk in patients with T2D. However, thiazolidinediones should be used cautiously because of their side effects on the heart, as is explained in the next paragraphs (Table 1).

## 3. Efficacy—Newer Oral Glucose-Lowering Drugs

### 3.1. DPP-4 Inhibitors

DPP4-Is are one of the main classes of drugs chosen for the management of T2D. They inhibit the enzyme (dipeptidyl peptidase 4) DPP4, increasing GLP1 and GIP levels. As a consequence of this, the release of insulin increases and the control of blood glucose levels is more effective [19].

DPP4-Is are increasingly used because of their ease of use and their safety compared to previous glucose-lowering drugs. Firstly, they control blood glucose levels, maintaining a neutral effect on body weight and, in addition, they would seem to have several other beneficial effects [20]. Different studies have shown that these drugs led to a decrease in high triglyceride levels, LDL-C, and free fatty acids, which are involved in atherosclerosis pathogenesis and are recognised as one of main cardiovascular risk factors. Moreover, their mild favourable effect on hypertension, valvular sclerosis, and endothelial function in patients with T2D has also been proven [21].

The latest studies also highlight the impact that DPP4-Is have on the modulation of the inflammatory pathway and on endothelial disfunction, because they are capable of minimising oxidative stress and inflammatory markers [22]. Moreover, as DPP4-Is have different targets, the positive effects on the heart and blood vessels may be due either to their action on the GLP-1-dependent pathway or to their action on an alternative pathway. However, the cardioprotective effect of DPP4 inhibitors is yet to be proven and further evidence is needed [19].

### 3.2. GLP-1 Agonists

GLP-1 is a peptide hormone released by gut endocrine cells that increases, on a glucose-dependent basis, the secretion of insulin and decreases glucagon levels [21,23]. Its actions are mediated by a specific receptor, the GLP-1 receptor, located not only in the gut, and on alpha and beta cells in the pancreas, but also in the heart, lungs, blood vessels, and central nervous system (CNS) [16]. GLP-1 exerts a pleiotropic effect on the cardiovascular system, upon which it acts via a direct or indirect pathway. Particularly, GLP-1 receptors are also located on atrial cardiomyocytes and on muscle cells of coronary arteries; GLP-1 would seem to be responsible for the increase in heart rate and coronary flow and, consequently, for the uptake of glucose by myocardiocytes.

Additionally, it promotes the cardiovascular well-being thanks to its role in the following areas:

(1) The gastrointestinal system, slowing motility and reducing the fatty acid absorption [24];
(2) The central nervous system (CNS), inducing satiety and thus weight loss, and the reduction in adipose tissue;
(3) The kidney, promoting natriuresis and reducing the volume of circulating, blood and in this way improving cardiac contractility;
(4) The immune system, reducing the state of inflammation and oxidative stress.

Therefore, given the multiple benefits that this hormone can lead to, drugs such as GLP-1 R agonists were firstly developed for the treatment of T2D and obesity. Moreover, these pharmacological agents contribute to CV health both directly and indirectly because they reduce CV risk factors such as diabetes and obesity, and also have a positive impact on the kidney [23,24].

The main drugs belonging to this class used are semaglutide, albiglutide, dulaglutide, exenatide, liraglutide, and lixenatide. They are classified on basis of their pharmacokinetic properties into short-acting (exenatide twice daily; lixenatide and oral semaglutide once daily) and long-acting drugs (liraglutide, semaglutide, exenatide, and dulaglutide once weekly). The first class act to reduce blood glucose levels after the meal, delaying gastric emptying. The long-acting class, instead, leads to an increase in insulin secretion both after the meal and in interprandial phases. For this reason, the latter class presents greater tolerability, less glycaemic fluctuations, and better adherence to therapy by patients [25,26].

Several studies demonstrated the effectiveness of these drugs in quickly decreasing HbA1c in patients with T2D, without any risk of hypoglycaemic episodes, with an effect on body weight loss. Moreover, GLP-1 agonists can be used in a standardised way compared to insulin and for all of these reasons the American Diabetes Association and European Association for the Study of Diabetes recommend using these pharmacological agents when oral antidiabetic agents alone fail (for example metformin), instead of insulin, despite the two classes having a similar efficacy in controlling blood glucose levels [26].

The multicentric and randomised study, SUSTAIN 10, compared the efficacy of once-weekly semaglutide, which is easier to manage, to that of daily liraglutide. It showed that once-weekly semaglutide led to a greater reduction in HbA1C and in body weight, especially in subjects with T2DM not controlled by 1-3 oral drugs [27].

In addition, a pooled analysis of SUSTAIN 6 and LEADER highlighted how semaglutide and liraglutide also have an important protective effect on the kidney, leading to a reduction in albuminuria and a slower decline in the Estimated Glomerular Filtration Rate, primarily in patients with a preexisting chronic renal disease [28]. Liraglutide is also capable of interdicting the development of atherosclerotic plaque, down-regulating acetyl-CoA acetyltransferase 1 (ACAT1), and in this way suppressing macrophage foam cell formation [29].

The same drugs can be employed for managing obesity, both in patients with diabetes and without it, because various studies have revealed that, also in patients without diabetic disease, GLP1-R agonists are effective in promoting weight loss in relation to a placebo [30].

Using these medications and changing one’s lifestyle are recommended both to obese adults and to adolescents to obtain greater and longer-lasting weight loss [31,32,33]. The recourse to GLP-1 R agonists may also be considered to contrast the recovery of body weight after bariatric surgery because they can lead to a consistent loss of gained weight [34].

### 3.3. SGLT2-Inhibitors

SGLT-2 inhibitors represent a new class of glucose-lowering drugs that reduce blood glycaemic levels, stopping glucose reabsorption in renal tubules, regardless of the insulin action. They have different beneficial metabolic and hemodynamic effects, which can improve cardiovascular prognosis [16,19,35].

Firstly, they cause body weight loss because of the increased glycosuria. Reducing blood glucose levels, they determine a reduction in glucotoxicity and thus, a greater insulin sensitivity [36].

Secondly, SGLT-2 inhibitors act also to decrease blood pressure values, as a consequence of their diuretic and natriuretic power [37]. Additionally, they improve the lipidic profile and decrease uric acid blood levels because of their uricosuric effect [36,38].

The combination of these actions determines better glycaemic control and an improved insulin sensitivity, which decrease the risk of cardiovascular events in patients with T2DM and insulin resistance.

SGLT-2 inhibitors can lead to further benefits such as a reduction in inflammatory response and oxidative markers, a reduction in collagen production implicated in cardiac remodelling, and the decrease in sympathetic overdrive, as shown in different experimental models [16,39,40].

They can also increase ketone bodies’ production, used as alternative energy source that is more efficient than glucose in generating ATP, which improve the insulin sensitivity of the heart and its function; deepening understandings of this topic could be beneficial in the future especially for patients with heart failure [41]. Finally, these drugs act on the kidney, decreasing the deterioration of renal function, which represents itself a risk factor for CV events [42]. The knowledge of SGLT-2 inhibitors’ effectiveness results, nowadays, in the increasing use of these pharmacological agents as monotherapy or associated with other antidiabetic drugs both in patients with T2D and with HF (Table 2).

## 4. CV Tolerability Profile

### 4.1. Established Oral Glucose-Lowering Drugs

#### 4.1.1. Metformin and Thiazolinedion

Metformin is a drug not only safe in terms of cardiovascular risk, but it is also effective in reducing it and promoting cardiovascular health. Several observational, database, and case–control studies show that metformin decreases the risk of Major Adverse Cardiovascular Events (MACEs) in diabetic patients with CVD. However, large-scale evidence for new antidiabetic oral drugs is still lacking [2,43]. On the other hand, thiazolinediones are a class of glucose-lowering drugs that need greater attention in terms of their use, because of their side effects. Several lines of evidence highlight that rosiglitazone may increase the risk of developing heart failure [44].

Similarly, in the PROactive trial (PROspective pioglitAzone Clinical Trial In Macrovascular Events) on patients with diabetic diseases and macrovascular diseases, the occurrence of HF was significantly greater in patients receiving pioglitazone compared to patients receiving a placebo [45].

Nevertheless, both via this trial and via the IRIS trial, conducted in same way on patients with T2DM, it emerged that patients in therapies with thiazolinediones had a lower risk of recurrence of combined endpoints consisting of stroke and MI [46]. 

#### 4.1.2. DPP-4 Inhibitors

Clinical trials regarding this class of pharmacological agents examined the incidence rate of MACEs, such as myocardial infarction, non-fatal stroke, death from cardiovascular causes, hospitalisation for instable angina, HF, and coronary revascularization, and documented the absence of statistically significant differences in the group of patients treated with DDP-4 inhibitors compared with patients taking placebo [47,48,49].

In addition, a network meta-analysis (NMA) of CVOTs on new antidiabetic drugs revealed that there were no significant differences among different drugs of this class with regard to all of the CV endpoints, and that they had a safety profile similar to placebo [50]. Specifically, the randomised, double-blinded trial, TECOS, studied the effects of sitagliptin. A total of 14,671 diabetic patients were enrolled and one group received sitagliptin and the other one placebo, in addition to their previous therapy. After a follow-up of 3 years, the primary composite endpoint and the rate of hospitalisation for HF was similar in the two groups, demonstrating the non-inferiority of sitagliptin. There was also no significant difference in terms of acute pancreatitis and pancreatic cancer [51].

SAVOR TIMI 53 was a trial which involved 1,692 patients with diabetes who had had a high risk of CVD, whose aim was to establish the safety of saxagliptin. After a period of observation of two years, patients treated with saxagliptin did not have a greater incidence of MACEs than patients who took the placebo, and the treated patients had a greater rate of incidence of hospitalisation for HF. Thus, even if saxagliptin improves glycaemic control, different approaches are needed to reduce cardiovascular risk [47].

Similarly, alogliptin was investigated in the trial EXAMINE, on patients with T2D and instable angina in the previous 90 days. In the same way, patients who took alogliptin did not have a greater rate of cardiovascular events or side effects such as hypoglycaemia, cancer, or pancreatitis, and they presented lower levels of HbA1C [49].

The safety of linagliptin was studied in two different trials, CARMELINA (Cardiovascular safety and Renal Microvascular outcomE study with LINAgliptin), which was conducted on diabetic patients with high CV risk (patients who showed albuminuria, macrovascular disease, or renal impairment) and which focused above all on the population with advanced CKD [52], and CAROLINA (Cardiovascular Outcome Trial of Linagliptin vs. Glimepiride in Type 2 Diabetes) [53], which involved the same kind of population, but it was the first and the only trial that made a head-to-head comparison of the use of DPP4 inhibitors and another class of glucose-lowering agents as sulphonylureas [19].

Finally, the safety profile of DPP4 inhibitors is excellent, especially in the elderly population or in subjects with renal impairment or high CV risk. However, there are still unresolved issues. For example, it should be clarified if the use of saxagliptin increases the risk of HF in patients with a high risk of CV events. Moreover, it is important to underline that in all of these trial, except in CARMELINA, patients with kidney disease were not included. Therefore, more consistent data are needed to understand the potential of these pharmacological agents.

#### 4.1.3. GLP-1 Agonists

GLP-1 agonists seem to be harmless in terms of cardiovascular side effects, and, at same time, they promote cardiovascular health. It was proven that linaglutide, semaglutide, and albiglutide are capable of reducing the main major adverse cardiovascular effects, consisting of death cause by CV, non-fatal MI, or stroke. On the other hand, lixisenatide and exenatide have a neutral impact on CV well-being [26]; however, the impact of albiglutide in terms of the reduction in MACEs is not very relevant [54].

Additionally, liraglutide not only contributes to decreasing the incidence of MACEs by 13% and death by 21%, but it seems to reduce all-causes mortality by 15% compared to the placebo in patients with CVD, as revealed by the LEADER trial [55]. Similarly, the REWIND STUDY enrolled both patients with a previous history of atherosclerotic blockage and without it, demonstrating that it leads to a reduction in MACEs in the whole study population [56]. 

As shown in the meta-analysis of different randomised controlled trials, pharmacological agents belonging to this class of antidiabetic drugs can help to decrease the risk of new-onset HF. In addition, they are able to act on different pathways such as that of inflammation and atherosclerosis, and on blood vessel’s function, and they also have to be considered as important tools with which to correctly manage diabetic patients who are themselves a remarkable CV risk [25]. The only significant and most common side effects are gastrointestinal, which occur, above all, at start of therapy, and are registered frequently with the use of semaglutide, but in the majority of cases these episodes are self-limited and short [26].

#### 4.1.4. SGLT2-Inhibitors

Over the last two years, different RCTs on SGLT-2 inhibitors (empaglifozin, dapaglifozin, and canaglifozin) demonstrated the role they play in improving cardiovascular and renal endpoints, especially in terms of hospitalizations ad mortality for HF.

In a pooled analysis of all of RCTs, except for OUTCOME and CANVAS, a decrease in the incidence rate of myocardial infarction, hospitalisation for HF, and mortality for CV or other causes was registered in patients treated with this class of drug, with an improvement in CV outcomes and in survival in diabetic patients [57].

EMPAREG OUTCOME is a large trial focused on CV safety related to the use of empaglifozin in patients with T2DM and established CVD. It was found that empaglifozin led to a moderate but significant reduction in the primary composite CV endpoint (death for CV causes, non-fatal MI, and non-fatal stroke) and a remarkable decrease in CV and all-causes mortality [58]. Moreover, empaglifozin led to a significant decrease in hospitalisation or death for HF and these results arose quickly, probably because of its diuretic effect [59].

In addition, as CKD is considered a major cardiovascular risk factor, the impact of empaglifozin on the kidney could improve the further prognosis of patients with CV risk and diabetes. In fact, patients treated with empagliflozin showed a slower progression of renal disease and less clinical events compared with the patients taking a placebo [60].

CANVAS PROGRAM includes data from two different trials, CANVAS and CANVAS-R, on the use of canaglifozin. It involves diabetic subjects with or CVD or high cardiovascular risk. Similarly to what is seen for empaglifozin, the incidence of CV (triple MACE: death for CV causes, non-fatal MI, and non-fatal stroke) was significantly lower in patients treated with canaglifozin [61]. Likewise, the improvement in renal function, already registered in EMPAREG OUTCOME, was confirmed in this analysis. However, the use of this drug led to an increased risk of the amputation of the toes.

Finally, DECLARE TIMI-58 is a meta-analysis of RCTs on dapaglifozin, focused on the occurrence of CV events. It highlights that dapaglifozin is not associated with an increase in CV events and demonstrates a potential positive CV effect on patients with T2D in primary CV prevention. This data applies for patients with different features, such as different cardiovascular risk, age, type, and number of events, type, and number of risk factors. Nevertheless, the trial is still ongoing and from its results we will understand if dapaglifozin can also be used in patients with CDV in secondary prevention, and if it has different side effects from the other drugs of the same class [62].

The safety and effectiveness of this class of antidiabetic drugs were also analysed by a broad observational study, the CVD REAL (Comparative Effectiveness of Cardiovascular Outcomes in New Users of Sodium–Glucose Cotransporter-2 Inhibitors) which involved different European countries (Norwey, Denmark, Sweden, Germany, and the UK) and the USA. This study confirmed that subjects who used glifozines had a lower risk of hospitalisation for HF and for all-causes mortality compared to those who used other antidiabetic medications, extending the possibility of treating the entire diabetic population with them in real-life settings [63].

Although glifozines improve CV and renal outcomes, care should be taken to consider the common side effects, such as genital mycotic infections, ketoacidosis, and periferic amputation, which occur in patients with specific features [64] (Table 3).

## 5. Conclusions

Lifestyle changes are important factors in preventing and managing T2D and CVD [2]. The most important determinants of the onset and progression of both diabetic disease and cardiovascular disease are hypertension, obesity, cigarette smoking, and hypercholesterolemia [3]. Innumerable studies have clearly documented that we can modify individual risk factors with targeted interventions. Specifically, a reduction in cholesterol-LDL values, cessation of cigarette smoking, reduction in blood pressure values with selective drugs, and reduction in body weight have clearly documented effects in terms of reducing cardiovascular morbidity [6,7].

In fact, among these targeted interventions listed above, there is one that represents a key element in the prevention and treatment of cardiovascular disease and diabetes, which is often mentioned but not always implemented rigorously for various reasons, which is the reduction in body weight, with an appropriate diet. This simple and inexpensive intervention can directly modify both the reduction in plasma concentration of cholesterol values and blood pressure values [65,66].

Therefore, the recent introduction of hypoglycaemic drugs (liraglutide and semiglutide) capable of significantly modifying body weight has resulted in a reduction in both mortality and cardiovascular morbidity with excellent patient tolerability.

The multifactorial therapeutic approaches to diabetes and cardiovascular disease should therefore not disregard the use of these drugs given their significant effect on the various risk factors involved in the onset and progression of CVD. An important factor in the management of both T2D and CVD is body weight reduction.

The choice of glucose-lowering therapy is often influenced by effects on weight, when weight loss or avoiding weight gain is a priority. Insulins, sulphonylureas, and pioglitazone all cause weight gain; metformin, acarbose, and DPP-4 inhibitors are weight neutral or may result in small amounts of weight loss; and SGLT2 inhibitors and GLP-1 RAs are associated with clinically meaningful weight loss, with the weight effects of GLP-1 RAs being more pronounced than those of SGLT2 inhibitors.

SGLT-2 receptors are exclusively present on renal proximal convoluted tubules and are responsible for the reabsorption of almost all of the body’s glucose and the majority of the sodium filtered in the glomerulus. The inhibition of this receptor causes a diuretic effect causing the excretion of both sodium and glucose, presenting a non-insulin-dependent way of reducing serum glucose levels without increasing the risk of hypoglycaemia.

SGLT-2 inhibitors have emerged as novel therapies for the management of patients with chronic kidney disease. Their efficacy was initially observed in the CREDENCE trial, which primarily evaluated the reno-protective effect of SGLT-2 inhibitors, and where canagliflozin was found to significantly reduce major cardiovascular and renal events in patients with T2DM. Since then, these findings have been further validated by Dapagliflozin and Prevention of Adverse Outcomes in Chronic Kidney Disease (DAPA-CKD) [67], and The Study of Heart and Kidney Protection With Empagliflozin (EMPA-KIDNEY) [68] trials, demonstrating similar benefits irrespective of T2D. Given its wide-ranging effects on multiple organ systems, including the renal, cardiac, and endocrine systems, SGLT-2 are the pioneer metabolic drugs. SGLT-2 inhibition leads to only a modest reduction in haemoglobin A1c when used for the management of T2D; however, it still leads to a significant reduction in cardiovascular outcomes in these patients, which indicates that adverse cardiovascular events in T2D may not be solely mediated by dysglycaemia. Moreover, SGLT-2 inhibitors are the only drug effective across the entire LVEF spectrum of HF, through postulated mechanisms that are not limited to the diuretic or natriuretic effects of the drug.

GLP-1 RA and SGLT-2 inhibitors are the most recent addition to drug therapies that reduce morbidity and mortality in T2D and HF, and are effective across the spectrum of LVEF. The mechanisms underlying these benefits are not well-established, with current ongoing research suggesting a vast number of metabolic and biomolecular targets that may play a role in incurring cardio-renal-metabolic effects in HF. The efficacy of these drugs extends across chronic kidney disease, and it is imperative to ensure the appropriate implementation of SGLT-2 inhibitor therapy use across these populations to improve event-free survival and reduce the associated healthcare costs.

These latter indications are essential to the choice of pharmacological treatment of both T2D and CVD and complications. Greater attention should be given to all those active ingredients that have clearly documented both greater efficacy and better tolerability. The new key in hypoglycaemic treatment should adhere to these simple directions in order to ensure that the patient receives the best treatment in terms of both efficacy and tolerability (Table 4).

## 6. Final Thoughts

This paper is a narrative review rather than a systematic analysis and presents many limitations. In particular, the evidence was selected for relevance rather than through a formal search strategy. For these reasons, future works should integrate the multidisciplinary aspects of diabetes and cardiovascular disease management, including clinical pathways and long-term outcome data.

## Figures and Tables

**Table 1:** Principal effects of oral glucose-lowering drugs.

Metformin	Thiazolinedion	DPP-4 Inhibitors	GLP-1 Agonists	SGLT2-Inhibitors
- reduces glucose liver production - activates AMP-activated protein kinase the production of endothelial nitric oxide synthase (eNOS) - reduces oxidative stress, inflammatory response and platelet aggregation	- reduces insulin resistance - increase insulin sensitivity - reduce the level of circulating triglycerides and cholesterol low-density lipoprotein (C-LDL) and oxidative stress	- inhibit the enzyme (dipeptidyl peptidase 4) DPP4 - increasing GLP1 and GIP levels. - increased release of insulin and a more effective control of blood glucose levels.	- mimic the action of endogenous GLP-1, activating GLP-1R, thereby enhancing insulin secretion, inhibiting glucagon release, delaying gastric emptying, and reducing food intake through central appetite suppression.	- inhibit renal glucose reabsorption by blocking the SGLT2 cotransporters in the proximal tubules and causing glucosuria - reduces glycemia and lowers HbA1c - the accompanying sodium excretion reverts the tubuloglomerular feedback and reduces intraglomerular pressure

**Table 2:** Effects on cardiovascular mortality.

Metformin	Thiazolinedion	DPP-4 Inhibitors	GLP-1 Agonists	SGLT2-Inhibitors
**Reduction in CV** **mortality** - reduce inflammation - reduce hypertrophy - reduce fibrosis - reduce heart stiffness	**Increased risk in CV mortality** - increase hypertrophy - increase inflammation - increase cardiac fibrosis	**Neutral benefit in CV mortality** - reduce necrosis - increase apoptosis - reduce oxidative stress - reduce hypetrophy - increase myocardial perfusion	**Reduction in CV** **mortality** - reduce inflammation - reduce ROS - reduce blood pressure - reduce heart rate - increase myocardial contractily	**Reduction in CV** **mortality** - reduce hypertrophy - increase dyastolic function - reduce fibrosis - reduce epicardial fat - reduce glucose utilization

**Table 3:** Cardiovascular and renal effects.

**SGUT2 inhibitors** Empaglifoxin—dapaglifozin—canaglifozin	**Cardiovascular effects:** - Reduction in heart failure (hospitalizations and progression) - Reduction in cardiovascular mortality in high-risk patients (e.g., EMPA-REG OUTCOME) - Favorable effects also in non-diabetic patients with heart failure (e.g., DAPA-HF) **Renal effects:** - Slow down the progression of diabetic nephropathy - Reduction in albuminuria - Studies such as CREDENCE and DAPA-CKD have also demonstrated benefits in moderate-to-severe CKD
**GLP-1 receptor agonists** (es. liraglutide, semaglutide, dulaglutide)	**Cardiovascular effects:** - Reduction in MACE (Major Adverse Cardiovascular Events: heart attack, stroke, CV death) - Liraglutide (LEADER) → ↓ MACE - Semaglutide(SUSTAIN-6) → ↓ heart attack and stroke - Favorable effects on body weight and blood pressure **Renal effects:** - Indirect renal protection (↓ albuminuria, ↓ pressure) - Not as effective as SGLT2i in slowing the progression of CKD, but still useful
**Metformin**	**Cardiovascular effect:** - Historically considered neutral or moderatelyfavorable in temms of cardiovascuar risk - The UKPDS study showed a reduction inCVrisk in obese patients **Renal effects:** - It has no direct protective effects on the kidneys, but is well tolerated if renal functionis preserved (eGFR > 30)
**DPP-4 inhibitors** (e.g., sitagliptin, linagliptin)	**Cardiovascular effects:** - Cardiovascuar safe (e.g., TECOS, SAVORTIMI) **Renal effects:** - No significant benefits in terms of CV or renalrisk reduction

**Table 4:** Molecular targets.

**SGUT2 inhibitors**	Work by blocking the sodium-glucose cotransporter 2 (SGLT2) protein in the kidney’s proximal convoluted tubule, which prevents glucose from being reabsorbed into the blood and causes it to be excreted in the urine(glycosuria). This mechanism lowers blood, glucose levels, is independent of insulin, and can also lead to weight loss and a reduction inblood pressure, with benefits observed for heart and kidney disease progression.
**GLP-1 receptor agonists**	work by mimicking Glucagon-Like Peptide-1(GLP-1), a naturally occurring hormone, to activate GLP-1 receptors in the brain and pancreas. In the brain, they reduce appetite and increase feelings of fullness (satiety). In the pancreas, they stimulate glucose-dependent insulin release and suppress glucagon secretion, improving blood sugar control. Additionally, they slow gastric emptying, which further contributes to reduced food intake and improved glvcemic control.
**Metformin**	suppresses gluconeogenesis by inhibiting a mitochondrion-specific isoform of glycerophosphate dehydrogenase (mGPD). This, in turn, decelerates the dihydroxyacetone phosphate (DHAP)-glycerophosphate shuttle (glycerophosphate is also called glycerol-3-phosphate[G3P]).
**DPP-4 inhibitors**	work by blocking the dipeptidyl peptidase-4 (DPP-4) enzyme which prevents the breakdown of incretin hormones like glucagon-like peptide-1 (GLP-1) and glucose-dependent insulinotropic polypeptide (GIP). This action increases the levels of active incretins in the body, leading to increased glucose-dependent insulin secretion from the pancreas and suppressed glucagon release.

## Data Availability

No new data were created or analyzed in this study. Data sharing is not applicable to this article.
